# Relationship between institutional intensive care volume prior to the COVID-19 pandemic and in-hospital death in ventilated patients with severe COVID-19

**DOI:** 10.1038/s41598-022-26893-6

**Published:** 2022-12-24

**Authors:** Shunsuke Amagasa, Masahiro Kashiura, Hideto Yasuda, Mineji Hayakawa, Kazuma Yamakawa, Akira Endo, Takayuki Ogura, Atsushi Hirayama, Hideo Yasunaga, Takashi Tagami

**Affiliations:** 1 Division of Emergency and Transport Services, National Center for Child Health and Development, 2-10-1, Okura, Setagaya-ku, Tokyo, 157-8535 Japan; 2 Department of Emergency and Critical Care Medicine, Saitama Medical Center, Jichi Medical University, 1-847, Amanuma-cho, Omiya-ku, Saitama-shi, Saitama 330-8503 Japan; 3 Department of Emergency Medicine, Hokkaido University Hospital, N14W5, Kita-ku, Sapporo, 060-8648 Japan; 4 Department of Emergency Medicine, Osaka Medical and Pharmaceutical University, 2-7 Daigakumachi, Takatsuki, Osaka 569-8686 Japan; 5 Trauma and Acute Critical Care Center, Tokyo Medical and Dental University Hospital, 1-5-45 Yushima, Bunkyo-ku, Tokyo, 113-8519 Japan; 6 Department of Emergency Medicine and Critical Care Medicine, Tochigi Prefectural Emergency and Critical Care Centre, Imperial Foundation Saiseikai Utsunomiya Hospital, 911-1 Takebayashi-machi, Utsunomiya, Tochigi 321-0974 Japan; 7 Public Health, Department of Social Medicine, Graduate School of Medicine, Osaka University, 2-2 Yamadaoka, Suita, Osaka 565-0871 Japan; 8 Department of Clinical Epidemiology and Health Economics, School of Public Health, The University of Tokyo, 7-3-1 Hongo, Bunkyo-ku, Tokyo, 113-8654 Japan; 9 Department of Emergency and Critical Care Medicine, Nippon Medical School Musashikosugi Hospital, 1-396 Kosugimachi, Nakahara-ku, Kawasaki, Kanagawa 211-8533 Japan

**Keywords:** Medical research, Outcomes research

## Abstract

We aimed to evaluate the association between ICU patient volume before the COVID-19 pandemic and the outcomes of ventilated COVID-19 patients. We analyzed ventilated patients with COVID-19 aged > 17 years and enrolled in the J-RECOVER study, a retrospective multicenter observational study conducted in Japan between January and September 2020. Based on the number of patients admitted to the ICU between January and December 2019, the top third institutions were defined as high-volume centers, the middle third ones as middle-volume centers, and the bottom third ones as low-volume centers. The primary outcome measure was in-hospital mortality. Multivariate logistic regression analysis for in-hospital mortality and ICU patient volume was performed after adjusting for multiple propensity scores. Among 461 patients, 158, 158, and 145 patients were admitted to low-volume (20 institutions), middle-volume (14 institutions), and high-volume (13 institutions) centers, respectively. Admission to middle- and high-volume centers was not significantly associated with in-hospital death compared with admission to low-volume centers (adjusted odds ratio, 1.11 [95% confidence interval (CI): 0.55–2.25] and adjusted odds ratio, 0.81 [95% CI: 0.31–1.94], respectively). In conclusion, institutional intensive care patient volume prior to the COVID-19 pandemic was not significantly associated with in-hospital death in ventilated COVID-19 patients.

## Introduction

The coronavirus disease 2019 (COVID-19) pandemic caused by severe acute respiratory syndrome coronavirus type 2 (SARS-CoV-2) has become a major burden on health care systems worldwide^1^. One-fifth to one-third of hospitalized patients with COVID-19 become critically ill, and two-thirds of critically ill patients require invasive mechanical ventilation^2–5^. The case-fatality rate for these patients on mechanical ventilation has been reported to be approximately 45%^6^. The COVID-19 pandemic has resulted in an extreme increase in demand for intensive care unit (ICU) resources. Improvements in the health care system and survival rates for severe COVID-19 are urgent issues.

An increase in the number of medical and surgical patients improves the prognosis of patients, especially those who are critically ill or have undergone complex surgical procedures^7,8^. Many studies suggest that ICU patient outcomes are related to the number of patients treated and the number of beds available^8–14^. Therefore, critically ill patients may be best treated in the ICU, where more high-risk patients are treated. However, there are also reports that the volume-outcome relationship was not observed^15–19^ and that patient outcomes are worse in hospitals with a high number of ventilated patients or high ICU occupancy rate^20,21^. Few studies have examined whether the outcome of COVID-19 ICU patients is better in hospitals that are accustomed to treating a high volume of ICU patients. The examination of the volume-outcome relationship in terms of the usual number of ICU patients and severe COVID-19 may help improve the care, outcomes, and health care system of COVID-19 ICU patients.

We hypothesized that routine treatment of a large number of ICU patients and proficiency in ICU care would be associated with better outcomes in COVID-19 ICU patients. To evaluate our hypothesis, we investigated the association between institutional ICU patient volume before the COVID-19 pandemic and outcomes in ventilated ICU COVID-19 patients.

## Methods

### Study design and setting

The current study was one of the data analyses from the J-RECOVER study. The J-RECOVER study was a retrospective multicenter observational study conducted in Japan between January and September 2020. The design, data collection methods, and protocols of the J-RECOVER study have been previously reported in detail^22^. The current study was performed in accordance with the Declaration of Helsinki. The present study was approved by the institutional review boards of Nippon Medical School Musashikosugi Hospital (representative institution, application number:561-2-26), and the requirement for informed consent was waived because of the anonymous nature of the data used. Treatment protocols were left to each institution.

### Patients

The J-RECOVER study included COVID-19 patients admitted to the participating institutions during the study period. The present study included ventilated patients with severe COVID-19 aged > 17 years and were enrolled in the J-RECOVER study. Patients were excluded if they had missing data on the number of ICU admissions in 2019 at the institution.

### Data collection

The patients’ clinical information was obtained from diagnosis procedure combination (DPC) data and medical records. The DPC system is comprehensively evaluated for acute inpatient care costs. Administrative billing data have been created and stored electronically at each institution under a comprehensive payment system based on DPC^22,23^. DPC data included sex, date of birth, the primary purpose of care during hospitalization, date of admission, date of discharge, transportation of the patient, route of admission, hospital referral or outpatient department admission, scheduled or emergency care admission, emergency ambulance transport, discharge destination, and the outcomes of discharge. In addition, other necessary information that was not available from the DPC data was obtained from the institutions’ medical records and personnel.

### Measurements and definitions

The primary outcome measure was in-hospital death due to COVID-19 during hospitalization. Intensive care volume was defined as the number of patients admitted to the ICU between January and December 2019, the year before the COVID-19 pandemic, and it was subdivided into three categories, as per previous studies^12,24,25^ to evaluate the dose–response relationship. A cutoff number was defined to determine the number of patients as evenly as possible. The top third institutions were defined as high-volume centers, the middle third ones as middle-volume centers, and the bottom third ones as low-volume centers. We used the Charlson comorbidity index to measure underlying disease^26^. We used the Sequential Organ Failure Assessment (SOFA) score as an index of severity^27^.

### Statistical analysis

Demographic factors, patient characteristics, hospital care, and outcomes were compared among patients treated in high-, middle-, and low-volume centers. We analyzed trends in discrete variables among the three groups using the Mantel–Haenszel trend test and analyzed continuous variables using the Kruskal–Wallis test.

Regarding missing values, we performed multiple imputations^28^. Twenty multiple imputed datasets were generated. We performed multivariate logistic regression analyses to evaluate the association between ICU case volume and in-hospital death in each dataset, and the estimates were then combined.

Multiple propensity score analysis was used in the multivariate analysis to adjust for measurable prehospital confounding factors^29,30^. The propensity score indicates the conditional probability of a specific exposure given a series of measured baseline variables and is a well-established method for reducing confounding effects in observational studies. The propensity score is used to compare two groups; however, the multiple propensity score was used since this was a comparison of three groups^30–32^. Moreover, instead of matching and IPTW, which are frequently used in the comparison of two groups using propensity scores, multiple propensity scores were used as covariates in the multivariate analysis to adjust for confounding factors since this is a three-group comparison. A multiple propensity score is the conditional probability of being classified into three or more specific groups, given a set of observed baseline covariates. We adapted a multinomial logistic regression model to estimate the multiple propensity score for the present analysis. The model fell into one of the three groups along with hospital characteristics, including teaching status, number of hospital beds, number of ICU beds, patient demographics (age, sex, and Charlson comorbidity index), and prehospital factors (number of days from symptom onset to hospitalization, number of days from positive polymerase chain reaction test to hospitalization, transfer from another hospital, and SOFA score). Multivariate logistic regression was then used to analyze the association between ICU volume and in-hospital mortality, adjusting for multiple propensity scores and in-hospital variables such as favipiravir, remdesivir, ICU admission, use of prone position, and use of extracorporeal membrane oxygenation (ECMO), while also adjusting for within-hospital clustering using a generalized estimating equation.

All statistical analyses were two-sided, and a level of 0.05 was considered statistically significant. We analyzed the data using SPSS statistical software version 26 (IBM, New York, USA).

## Results

### Patients

**Figure 1:**
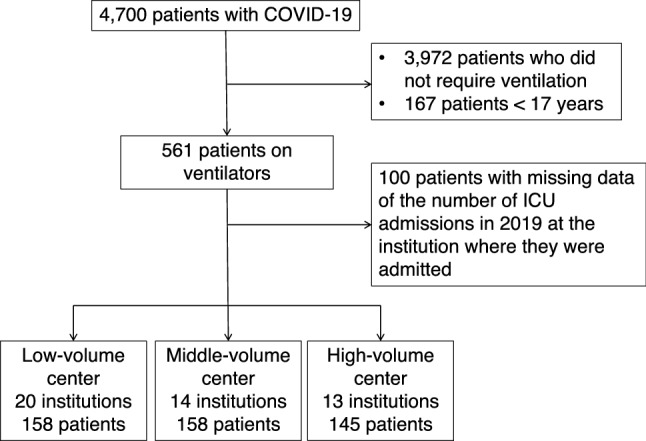
Flowchart of the study.

The J-RECOVER study enrolled 4,700 patients with COVID-19 from 66 institutions. Among them, 561 patients aged > 17 years required ventilator management (Fig. 1). After excluding 100 cases with missing data on the number of ICU admissions in 2019 at the institution where they were admitted, 461 patients were included in the analyses. A total of 158, 158, and 145 patients were admitted to low-volume (20 institutions), middle-volume (14 institutions), and high-volume (13 institutions) centers, respectively. There were no ventilated patients in 11 institutions, and there were no data on the number of ICU admissions in 2019 in 8 institutions that had ventilated patient admissions.

### Institutional information and patient characteristics

There were no significant differences in the number of academic hospitals, hospital beds, patients per institution, and ECMO patients per institution among the three groups (Table 1). Higher ICU case volume showed a significant tendency of higher ICU beds (< 0.001).

**Table 1:** Institutional information.

	Low-volume center (n = 158)	Middle-volume center (n = 158)	High-volume center (n = 145)	*p*
Number of institutions	20	14	13	
Number of academic hospitals, n (%)	10 (50.0)	6 (42.9)	9 (69.2)	0.34
Number of hospital beds, median (IQR)	744 (551–977)	659 (580–737)	800 (505–999)	0.30
Number of ICU beds, median (IQR)	16 (10–26)	31 (23–40)	42 (32–54)	< 0.001
Number of ICU admissions in 2019, median (IQR)^a^	775 (427–1036)	1963 (1775–2187)	3613 (2661–5475)	< 0.001
Number of patients per institution, median (IQR)	5 (3–11)	7 (3–15)	5 (3–16)	0.85
Number of ECMO patients per institution, median (IQR)	1 (0–2)	1.5 (1–3.5)	0 (0–3.5)	0.26
Number of ICU beds per emergency medicine specialist, median (IQR)	2.0 (1.4–5.2)	3.3 (2.1–4.6)	3.6 (2.7–5.8)	0.22
Number of ICU beds per intensivist, median (IQR)	3.4 (2.3–10)	8.7 (4.7–10.0)	7.7 (4.0–14.9)	0.10

*IQR* interquartile range, *ICU* intensive care unit.

^a^ICU admissions from January to December 2019.

Characteristics of the study population were described in Table 2. The proportion of men in the high-volume center was significantly higher than that of men in the other two groups (p = 0.04). Higher ICU case volume showed a significantly lower SOFA score on admission (p = 0.03). Patients in middle-volume centers had a lower probability of prone position (p < 0.001).

**Table 2:** Characteristics of the study population.

	Missing data	Low-volume center (n = 158)	Middle-volume center (n = 158)	High-volume center (n = 145)	*p*
Age, median (IQR)	0	66 (56–73)	69 (59–78)	66 (55–75)	0.05
Sex, male, n (%)	0	121 (76.6)	114 (72.2)	127 (87.6)	0.04
Transfer from another hospital, n (%)	0	91 (57.6)	85 (53.8)	86 (59.3)	0.14
Charlson comorbidity index	0				0.34
0, n (%)		94 (59.5)	113 (71.5)	91 (62.8)	
1, n (%)		39 (24.7)	33 (20.9)	34 (23.4)	
2, n (%)		16 (10.1)	5 (3.2)	14 (9.7)	
3, n (%)		5 (3.2)	3 (1.9)	3 (2.1)	
4 or more, n (%)		4 (2.5)	4 (2.5)	3 (2.1)	
Number of days from symptom onset to hospitalization, median (IQR)	11	8 (5–11)	7 (5–10)	8 (6–12)	0.39
Number of days from PCR test to hospitalization, median (IQR)	85	2 (1–6)	2 (1–7)	2 (0–5)	0.31
SOFA score on admission, median (IQR)	91	5 (3–8)	4 (2–8)	3 (3–6)	0.03
Favipiravir administration, n (%)	0	120 (75.9)	108 (68.4)	111 (76.6)	0.94
Remdesivir administration, n (%)	0	45 (28.5)	31 (19.6)	36 (24.8)	0.43
ICU admission, n (%)	0	149 (94.3)	151 (95.6)	145 (100)	0.07
Prone position, n (%)	32	69 (43.7)	38 (24.1)	58 (40.0)	< 0.001
ECMO, n (%)	0	35 (22.2)	36 (22.8)	21 (14.5)	0.10

*IQR* interquartile range, *PCR* positive polymerase chain, *SOFA* sequential organ failure assessment, *ICU* intensive care unit, *ECMO* extracorporeal membrane oxygenation.

### The differences in outcomes among institutions

There were no significant differences in transfer to another department, transfer to another hospital, length of stay in the hospital, length of stay in ICU, and in-hospital death among the three groups (Table 3).

**Table 3:** Different outcomes among institutions.

	Missing data	Low-volume center (n = 158)	Middle-volume center (n = 158)	High-volume center (n = 145)	*p*
Transfer to another department, n (%)	0	51 (32.3)	50 (31.6)	69 (47.6)	0.07
Transfer to another hospital, n (%)	2	72 (45.6)	69 (43.7)	50 (34.5)	0.05
Length of stay in hospital, day, median (IQR)	0	21.5 (11–35)	22.5 (14–21)	23 (13–34.5)	0.66
Length of stay in ICU, day, median (IQR)	19	11 (7–21)	14 (7–21)	11 (7–22)	0.24
In-hospital death, n (%)	0	40 (25.3)	47 (29.7)	35 (24.1)	0.84

*IQR* interquartile range.

### Multivariate logistic regression analyses for 1 month survival adjusted by multiple propensity score

Admission to middle-volume centers was not significantly associated with in-hospital mortality compared with admission to low-volume centers (adjusted odds ratio, 1.11 [95% confidence interval (CI): 0.55–2.25]. In addition, high-volume centers were not significantly associated with in-hospital mortality compared to admission to low-volume centers (adjusted odds ratio, 0.81 95% CI: 0.31–1.94) after adjustment for multiple propensity scores and in-hospital variables (Table 4).

**Table 4:** Multivariate logistic regression analyses for in-hospital death adjusted by multiple propensity score and in-hospital variables (n = 461).

	Crude OR (95% CI)	*p*	Adjusted OR (95% CI)	*p*
**Institution**				
Low-volume center	Reference		Reference	
Middle-volume center	1.25 (0.76–2.05)	0.38	1.11 (0.55–2.25)	0.78
High-volume center	0.94 (0.56–1.58)	0.81	0.81 (0.31–1.94)	0.58

A multiple propensity score was defined as the conditional probability of falling into a particular group of three groups of institutional case volume. We fitted a multinomial logistic regression model, which fell into one of the three groups as patient demographics and prehospital factors.

## Discussion

The current study evaluated the association between the institutional ICU patient volume before the COVID-19 pandemic and in-hospital mortality in ventilated COVID-19 patients. There was no significant association between the institutional ICU case volume in 2019 and in-hospital death after adjusting for patient characteristics, institutional information, and prehospital and in-hospital confounding factors. Since there was minimal bias in the proportion of academic hospitals, staffing ratio, advanced treatments such as ECMO, prone ventilation among the groups, size effect of result, and no dose–response relationship of volume-outcome, low-volume center may not worsen the outcome for severe COVID-19. The findings from the present study may be useful in developing a medical system for patients with severe COVID-19.

While many previous studies evaluated the volume-outcome relationships in critical care and found that high patient volumes may improve survival outcomes^8–14^, several studies found no association^15–19^. Some studies observed that high patient volumes may be associated with worse outcomes^20,21^. These variances among study findings may be due to differences in health care systems and hospital types, ICU management developments, or differences in statistical methods. What differentiated this study from previous volume-outcome relationship studies regarding the number of ICU patients treated and their outcomes is that it examined the association between the number of pre-pandemic ICU patients and the outcomes of COVID-19 ICU patients. In other words, our study examined whether institutions accustomed to ICU care can improve the outcomes of COVID-19 ICU patients. We hypothesized that severely ill COVID-19 patients would be best treated in ICUs that have treated higher-risk patients. However, contrary to our hypothesis, our study did not observe a significant association between the institutional ICU patient volume before the COVID-19 pandemic and in-hospital mortality.

Several reasons could explain why there was no significant association between institutional ICU patient volume and outcomes for COVID-19 ventilated cases. Many of the participating hospitals in this study were tertiary emergency centers. Both centers with high and low ICU patient volume in 2019 may have been habitually proficient in ICU care. There was minimal bias among the groups in staffing ratios of emergency medicine and intensive care specialists. In addition, the Ministry of Health, Labour and Welfare (MHLW) has set a standard of (at least) one nurse per two patients in all ICU. Advanced treatments such as ECMO and prone positioning were equally or more frequently performed at the smaller institutions. The availability of staff resources, even in smaller institutions, may be one of the factors that contributed to the absence of a volume-outcome relationship. The number of COVID-19 patients during the study period was also less biased among the three groups, suggesting that the ICU burden from COVID-19 was not unbalanced. In addition, ICU resources for severe COVID-19 were maintained in Japan during the study period^33^. In an ICU such as that in Japan, that is, a system in which there are more than a certain number of emergency physicians, intensivists, and nurses at the facility for each patient, there may not be a volume-outcome relationship. Further large-scale study is needed to evaluate this generated hypothesis.

Previous studies examining volume-outcome relationships for ICU care have included patients with varying diseases rather than patients with a specific disease or intervention^34^. The overall impact is therefore unclear. The study included COVID-19 patients who were on ventilator management, giving a relatively clear depiction of the patient population. Multiple propensity scores were also used to adjust for many confounding factors such as patient characteristics, institutional information, and prehospital and in-hospital factors. Few studies have examined the association between ICU patient volume prior to the COVID-19 pandemic and the outcomes of COVID-19 ICU patients. These results are considered important for constructing a health care system.

There were several limitations in the present study. First, this study did not have a large number of eligible institutions and cases. Thus, although this study did not find a significant difference between ICU patient volume and outcome, a volume-outcome relationship could not be ruled out. Further prospective large-scale studies are needed to assess the association between ICU patient volume and the outcomes of patients with severe COVID-19. Second, the J-RECOVER study was not a population-based study and was conducted at a volunteer hospital. The generalization of the results may be limited. Third, several patients were excluded due to missing ICU admission data in 2019. Since the data are per-institution, patients were missing on a per-institution basis, and the impact on the results is unknown.

## Conclusions

In the present study, institutional ICU patient volume prior to the COVID-19 pandemic was not significantly associated with in-hospital death in ventilated COVID-19 patients after adjusting for patient demographics, institutional information, and prehospital and in-hospital factors. In ICUs with adequate staff resources, small institution may not worsen the outcome. Further prospective large-scale study is needed to determine the association between institutional ICU patient volumes and severe COVID-19 outcomes.

## Data Availability

The datasets generated and/or analyzed during the current study are not publicly available because the use of the data was limited to institutions that participated in the registry. Datasets are available from the corresponding author upon reasonable request.
